# Low-Cost Recognition of Plastic Waste Using Deep Learning and a Multi-Spectral Near-Infrared Sensor

**DOI:** 10.3390/s24092821

**Published:** 2024-04-28

**Authors:** Uriel Martinez-Hernandez, Gregory West, Tareq Assaf

**Affiliations:** 1 Department of Electronic and Electrical Engineering, University of Bath, Bath BA2 7AY, UK; 2 Multimodal Interaction and Robot Active Perception (Inte-R-Action) Lab, University of Bath, Bath BA2 7AY, UK

**Keywords:** plastic recognition, near-infrared sensor, machine learning, low-cost sensors, principal component analysis

## Abstract

This work presents an approach for the recognition of plastics using a low-cost spectroscopy sensor module together with a set of machine learning methods. The sensor is a multi-spectral module capable of measuring 18 wavelengths from the visible to the near-infrared. Data processing and analysis are performed using a set of ten machine learning methods (Random Forest, Support Vector Machines, Multi-Layer Perceptron, Convolutional Neural Networks, Decision Trees, Logistic Regression, Naive Bayes, k-Nearest Neighbour, AdaBoost, Linear Discriminant Analysis). An experimental setup is designed for systematic data collection from six plastic types including PET, HDPE, PVC, LDPE, PP and PS household waste. The set of computational methods is implemented in a generalised pipeline for the validation of the proposed approach for the recognition of plastics. The results show that Convolutional Neural Networks and Multi-Layer Perceptron can recognise plastics with a mean accuracy of 72.50% and 70.25%, respectively, with the largest accuracy of 83.5% for PS plastic and the smallest accuracy of 66% for PET plastic. The results demonstrate that this low-cost near-infrared sensor with machine learning methods can recognise plastics effectively, making it an affordable and portable approach that contributes to the development of sustainable systems with potential for applications in other fields such as agriculture, e-waste recycling, healthcare and manufacturing.

## 1. Introduction

Plastics are widely used materials that can be found in a large variety of products in daily use such as food containers, bags, bottles, cards, telephones, computers and household objects [1,2]. The use of plastics for manufacturing of consumer products is attractive for the global economy since they are versatile, lightweight, low-cost, resistant to corrosion and durable [3,4]. However, the mass production, uncontrolled usage and low biodegradability of plastics have led to the generation of tons of waste contaminating the environment (streets, parks, cities, beaches, oceans), increasing carbon levels and contributing to health problems [5,6].

For this reason, recycling and reuse of plastics are urged for better waste management, cutting the manufacturing of new plastic objects and closing the material–consumption loop to maintain our natural resources and enable a circular and sustainable economy [7,8]. To facilitate the recycling process, the American Society for Testing and Materials (ASTM) has grouped plastics into seven main categories: (i) polyethylene-terephthalate (PE), (ii) high-density polyethylene (HDPE), (iii) polyvinyl-chloride (PVC), (iv) low-density polyethylene (LDPE), (v) polypropylene (PP), (vi) polystyrene (PS) and (vii) others [9]. However, efficient automated recycling and sorting processes require the identification of plastic waste, which is a critical task given that waste is usually mixed with different types of plastics and other objects [10]. Currently, plastics can be identified automatically by extracting data and analysing features of plastic waste using a variety of sensors and computational methods, including RGB cameras, hyperspectral imaging (HSI), near-infrared spectroscopy (NIR), visual image spectroscopy (VIS), and X-ray fluorescence (XFR) [11,12]. These sensors have shown their capability for accurate identification of plastics; however, most of them tend to be highly expensive (GBP 6k–50k) and accessible only to few groups, limiting their potential to tackle the worldwide issue of plastic pollution.

This work presents an alternative approach for recognition of plastic types using a low-cost near-infrared spectroscopy (NIR) sensor module (∼GBP 75) and a generalised pipeline with a set of machine learning (ML) methods. Our proposed approach is highly affordable compared to the traditional methods that use expensive spectroscopy sensors. The sensor module, which can perform measurements from the visible to NIR, is used together with an experimental setup to extract properties systematically from six (PET, HDPE, PVC, LDPE, PP, PS) different types of household plastic waste. The data collected are analysed using a set of ten computational methods including Multi-Layer Perceptron (MLP), Convolutional Neural Networks (CNNs), Linear Discriminant Analysis (LDA), AdaBoost, Random Forest, Decision Trees, Support Vector Machines (SVMs), Naive Bayes, Logistic Regression and k-Nearest Neighbour (kNN). The potential of these methods for plastic recognition is also evaluated by adding Principal Component Analysis (PCA) and LDA stages, which also offer a key and powerful preprocessing step for dimensionality reduction. The systematic data analysis and experimental results show that the low-cost NIR sensor module, with ML and dimensionality reduction of sensor data, can recognise plastic waste accurately and reliably while being affordable for any lab. Overall, the results show the potential of low-cost NIR spectroscopy sensors for material identification tasks and also their suitability for the design of affordable, reliable and portable tools that can contribute to reaching a circular and sustainable economy.

The rest of this paper is organised as follows: previous related works are described in Section 2; the low-cost sensor, plastic data and recognition process are presented in Section 3; the experiments and results are described in Section 4; and the discussion and conclusion of this work are presented in Section 5 and Section 6, respectively.

## 2. Related Work

The recognition of plastics has become a crucial task for recycling and reusing materials and alleviating the uncontrolled production of consumer products made of plastic. This is also an important to reduce the worldwide problem of plastic contaminating our streets, cities and oceans. For this reason, researchers have developed a variety of approaches combining different sensors and computational methods to classify plastic categories defined by the American Society for Testing and Materials.

Commonly, material property extraction from plastics for analysis and recognition has been performed using different sensing technologies, including XRF, Raman spectroscopy, NIR, miniaturised near-infrared (MicroNIR), laser-induced breakdown spectroscopy (LIBS), and hyperspectral computer tomography (CT) [11,12,13,14,15]. In the work presented by [16], hyperspectral CT sensing was used for data extraction, together with Artificial Neural Networks (ANNs) and SVMs, for the recognition of plastics, achieving 80% and 61% accuracies, respectively. The analysis in this work employed virgin plastics only; common plastics from household waste were not evaluated. Data measured with an industrial in-line hyperspectral camera were processed using PCA and k-Means for dimensionality reduction and clustering of different plastics [17]. The plastics were clustered correctly; however, the need for an industrial-level sensor makes this approach highly expensive and unsuitable for application in other environments. Spectroscopy sensing is one of the most popular techniques for recognition of plastics. Four different spectroscopy sensors: Fourier transform infrared spectroscopy (ATR-FTIR), NIR, LIBS, and XRF and a set of machine learning methods including SVM, kNN, LDA, PCA–SVM, PCA–kNN and PCA–LDA were used for the recognition of six plastic types (PET, HDPE, PVC, LDPE, PP, PS) from marine debris [12]. Mean accuracies of 70%, 91%, 97% and 99% were achieved by the combination of XRF and kNN, LIBS and LDA, NIR and LDA, and ATR-FTIR and LDA, respectively.

Video cameras and images have also been used for the detection and recognition of plastics based on the objects’ shape and colour [18,19,20,21]. Colour images were used together with SVM and YOLOv5 networks for detection and classification of plastics with accuracies of 94.7% and 95.2%, respectively [22,23]. These methods showed to be accurate; however, they focused mainly on the recognition of plastic bottles. Computer vision and recycling labels have also been used for classification of plastic waste [24,25]. These works employed PCA techniques and image processing for feature extraction and SVM for classification processes, achieving mean accuracies ranging from 96% to 97.3% for identification of PET and non-PET plastics. RGB cameras mounted in a microcontroller with CNN models were able to recognise four types of plastic with a 74% accuracy [26]. An approach consisting of micro-cameras and a Raspberry Pi computer was able to measure and identify signals from three plastic types (PET, HDPE, LDPE) using image processing techniques [27]. Machine vision and YOLOv3 were used in [28] for the identification of marine plastics with a mean accuracy of 98%. YOLO methods have gained popularity for plastic and general waste identification, using vision sensors with accuracies ranging from 65% to 94.5% [29,30]. These vision-based approaches have potential for autonomous identification and sorting of plastics using robotic platforms. However, their performance still depends largely on the quality of the images taken with cameras and on the environmental conditions, and they require a high computational power. Furthermore, these vision-based methods rely on the objects’ shape and colour only [21,31] and do not consider the composition of the plastic material, which is key for the recognition of actual plastic types. Transfer learning techniques with deep learning methods have allowed the recognition of plastics with an 89.87% accuracy under different environmental conditions, including domestic and outdoor environments [32]. Even though a reliable accuracy was achieved by this work, the analysis was performed using online databases only, limiting the robustness of this approach [33]. A deep learning approach consisting of CNN layers, feature transfer and dense connections was able to identify plastic waste with a 69% accuracy [34]. This value is lower compared to other methods but the transfer feature process allows it to recognise other waste types, including metals, glass and fabrics [35]. The use of deep learning methods and vision sensing technologies is gaining popularity for waste detection. However, their performance relies on vision data only, while material properties, extracted using spectral sensors, play a key role in the robust and reliable identification of plastics and other materials.

The spectroscopy-based methods described here have shown to be reliable for plastic recognition with accuracies ranging from 80.56% to 99% [36,37,38]. However, these works require specialised sensors that tend to be expensive (>GBP 25,000) and large, making them unaffordable and impractical [39,40]. Therefore, in this work, a new low-cost and small-sized triad spectroscopy sensor module is employed with a set of ML methods for the recognition of six different plastic types. This approach is affordable and accessible for the masses but also allows for the design of portable solutions for a variety of applications in different fields, including waste management, agriculture, monitoring and inspection, construction and manufacturing.

## 3. Methods

### 3.1. Multi-Spectral Sensor

**Figure 1:**
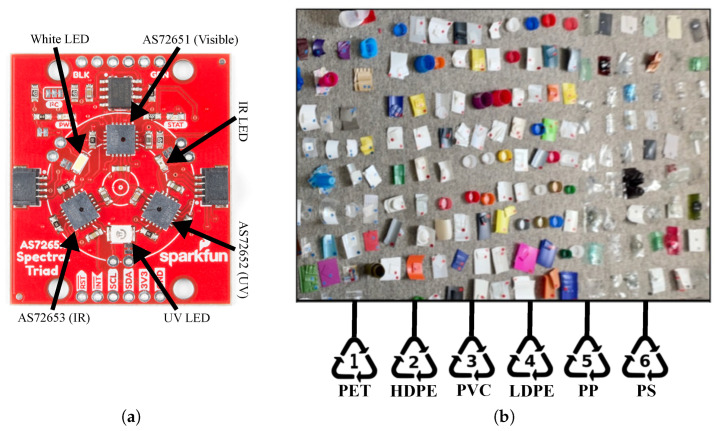
Low-cost spectroscopy sensor and examples of plastic waste. (**a**) Triad spectral sensor module from SparkFun Electronics [41]. (**b**) Examples of household plastic waste used for data collection and recognition processes.

This work examines the use of the low-cost multi-spectral chipset AS7265x from OSRAM. This chipset consists of three sensor devices, AS72651, AS72652 and AS72653, which can be used for spectral identification in a range from the visible to the NIR. Each of these three sensors includes six optical filters capable of measuring the light intensity in a range from 410 nm (ultraviolet—UV) to 940 nm (infrared—IR) with a full width at half maximum (FWHM) bandwidth of 20 nm. Thus, these three sensors were combined to form an AS7265x chipset with 18 multi-spectral wavelengths. These three multi-spectral sensors were integrated into a single package triad spectroscopy sensor, developed by the American company SparkFun Electronics, alongside visible, UV, and IR LEDs to illuminate and evaluate surfaces for light spectroscopy. This single package is used in the proposed experimental setup for plastic recognition. The sole unit cost of the AS7265x sensor is ∼GBP 15, while the integrated triad spectroscopy sensor is ∼GBP 56, which is ∼20x cheaper than high-cost spectroscopy sensors. Figure 1a shows the triad spectral sensor module with the arrangement of the visible, IR and UV chips and their corresponding LEDs.

### 3.2. Plastics Used for Data Collection

The majority of household waste comprises single or multiple types of plastics from the seven main categories identified by the American Society for Testing and Materials. These plastics and their corresponding identification codes are PET (code 1), HDPE (code 2), PVC (code 3), LDPE (code 4), PP (code 5), PS (code 6) and other (code 7). Therefore, for this work, typical plastics that can be found in household waste have been collected and labelled for the recognition process. Examples of plastic waste used in this work, such as pieces of bottles, lids and food containers are shown in Figure 1b. A total of 423 samples of plastic waste were employed for data collection, which include 100 LDPE, 100 HDPE, 100 PET, 100 LDPE, 15 PS and 8 PVC samples. All the plastics collected were washed at 60 °C to remove any surface debris, and a sample was cut from each plastic, ensuring it was as flat as possible, close to the dimensions of 50 mm × 50 mm for a systematic data collection process using the experimental setup described in Section 3.3. The samples were labelled with a unique ID according to their resin identification code (RIC) marked on the plastic item.

### 3.3. Experimental Setup for Data Collection

**Figure 2:**
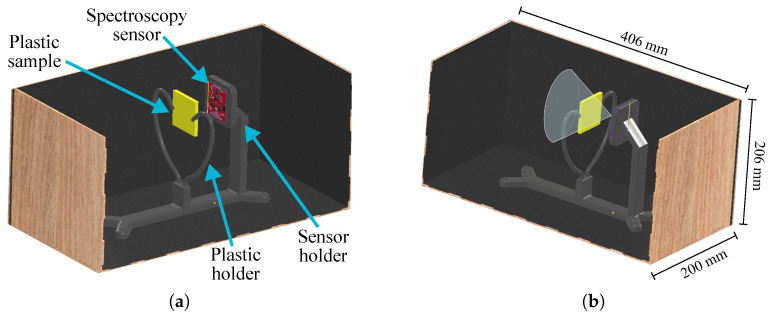
Illustration of the experimental setup used for systematic data collection. (**a**) View of the setup with the sensor and a plastic samples. (**b**) Setup with physical dimensions and illustration of the sensor measurement volume.

An experimental setup has been designed and developed by the authors of this work for systematic, reliable and robust data collection. This setup comprises a custom-built sealed wood-based box with dimensions of 406 mm × 206 mm × 200 mm and mounted 3D-printed holders to place the triad spectral sensor and plastic samples (see Figure 2). The whole experimental environment was painted in a thick matt black colour to reduce the effect of internal reflections. This setup also allows us to remove interference from external light sources and to keep a consistent background reflectance to ensure reliable measurements. The 3D-printed sensor and sample holder parts ensure that the triad spectral sensor module is mounted firmly with a consistent measurement distance and angle from the plastic sample. The plastic sample holder is mounted at a 50 mm distance from the sensor to allow the field-of-view divergence of the sensor to cover the whole surface of the sample with dimensions of 50 mm × 50 mm. Computer-aided design (CAD) files and different views of the experimental test environment with a plastic sample are shown in Figure 2a,b.

### 3.4. Data Collection

**Figure 3:**
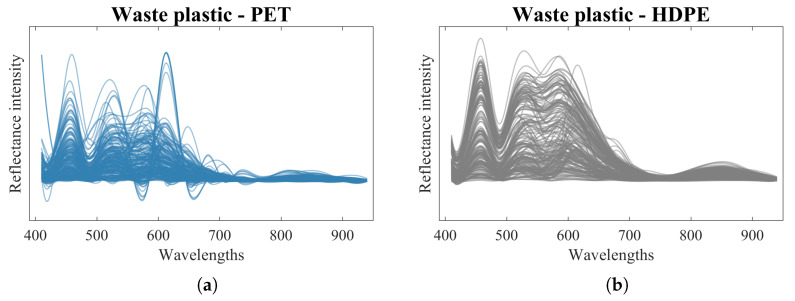

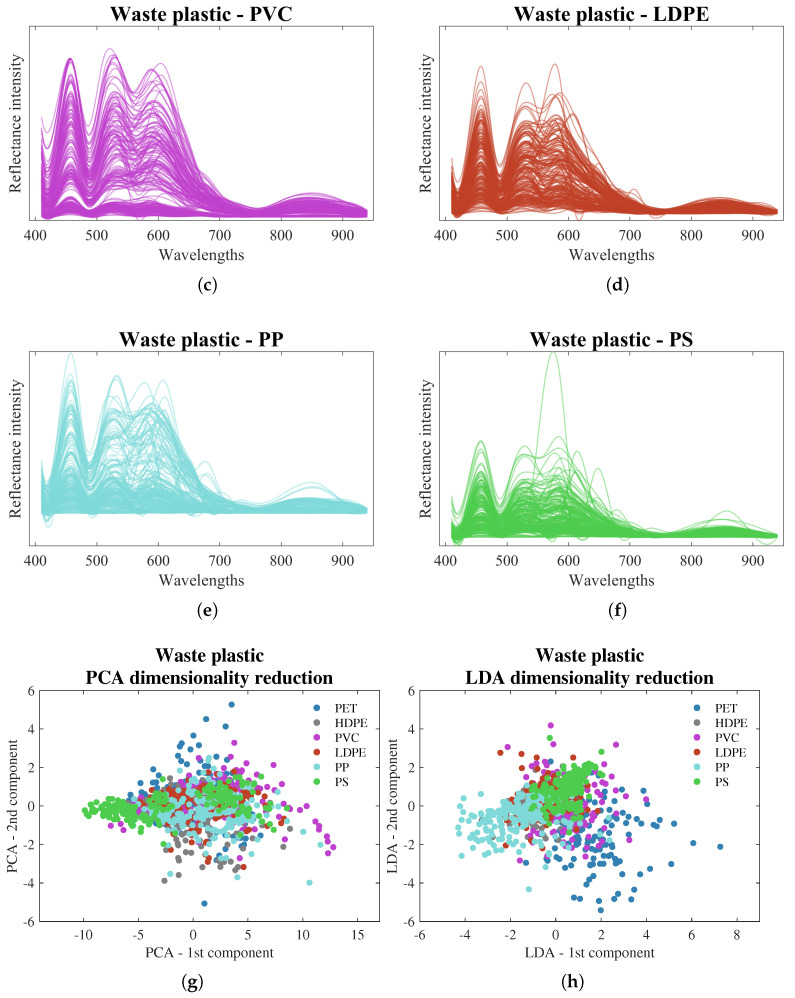
Example of data collected from plastic waste and visualisation with dimensionality reduction. (**a**–**f**) Spectral information with 200 measurements from each of the six plastic types. Two main components from the plastic waste using (**g**) Principal Component Analysis and (**h**) Linear Discriminant Analysis techniques.

The sensor and experimental setup shown in Figure 2 were used for systematic data collection from PET, HDPE, PVC, LDPE, PP, and PS plastic types. First, plastics obtained from household waste (see Figure 1b) were used for data collection. A total of 200 measurements from each plastic type were collected, creating a dataset of 1200 sensor readings. For PET, HDPE, LDPE and PP, two measurements were taken (200 measurements for each plastic), but for the case of PVC and PS, multiple measurements were taken to compensate for the lower number of plastic samples. Example plots with 200 measurements of raw spectra from each of the six types of plastic waste are shown in Figure 3a–f, where the *x* axis shows the range of wavelengths measured and the *y* axis shows the normalised amplitude of the signals. The datasets collected from plastic waste were used for training and testing the computational methods with the processing pipeline presented in Section 3.5 for recognition of plastic types.

### 3.5. Machine Learning Methods for Recognition

#### 3.5.1. Data Processing

The datasets with samples from all plastic waste types are split into training and testing groups for cross-validation processes as follows. First, the datasets are segmented into 10 folds, with 9 folds used to train each of the ML methods and the remaining fold for the test process. Then, the training and testing process is repeated 10 times, allowing each fold to be tested by each of the ML methods. Cross-validation has shown to be a robust approach for analysis of ML methods in a variety of recognition applications [42,43].

Second, a dimension reduction step is applied to the datasets using two different techniques: Principal Component Analysis (PCA) and Linear Discriminant Analysis (LDA). These techniques are widely used in recognition tasks in order to use relevant information from datasets, reducing the computational complexity and overfitting. Figure 3f,g shows plots with the two main components of the plastic waste dataset after applying PCA and LDA.

#### 3.5.2. Machine Learning Pipeline

**Figure 4:**
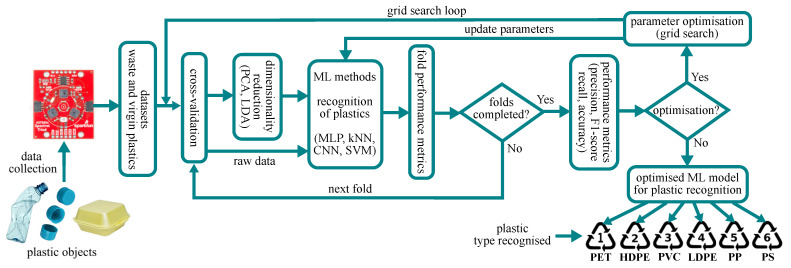
Generalised approach for implementation, training and testing of the set of machine learning methods composed of cross-validation, dimensionality reduction, recognition, performance metrics and optimisation stages.

Data collected from the triad spectroscopy sensor are used for recognition of plastic types using a set of computational methods. These methods include MultiLayer Perception (MLP), Convolutional Neural Networks (CNNs), Support Vector Machine (SVM), Decision Trees, Random Forest, AdaBoost, k-Nearest Neighbours (kNN), Logistic Regression, Linear Discriminant Analysis (LDA) and Naive Bayes, which have been chosen according to works from the literature about plastic recognition. As described in Section 3.5.1, the spectral data from the sensor module are also preprocessed applying PCA and LDA [44]. Thus, the combination of these two dimensionality reduction techniques and the set of computational methods (e.g., PCA–MLP, PCA–SVM, LDA–MLP, LDA–SVM) is also used for evaluation of the recognition performance. Figure 4 shows the diagram of the generalised and systematic methodology used to implement and optimise each computational method for recognition of plastics. This diagram is composed of five main processes: (1) cross-validation, (2) dimensionality reduction, (3) recognition, (4) performance metrics and (5) optimisation. The input dataset is split into training and testing folds for cross-validation to validate the performance of the set of ML methods. The folds are used for dimensionality reduction and then employed as input data for the recognition of plastic types. The output from the recognition process is used for the performance metrics and optimisation steps, which are described in Section 3.5.3 and Section 3.5.4.

#### 3.5.3. Performance Metrics

The performance of the computational methods used for the recognition process was evaluated using a set of metrics. These metrics include the accuracy of the identification of plastics, precision, recall and *F*1-score, which are commonly used for performance evaluations of ML methods [45,46,47]. The calculation of these metrics employs information from the correct recognition of the target class or true positive (TP), correct recognition of the non-target class or true negative (TN), incorrect recognition of the target class or false positive (FP) and incorrect recognition of a non-target class (FN). These metrics are calculated as follows:

(1) $$P=\frac{TP}{TP+FP}\times 100\%$$

(2) $$R=\frac{TP}{TP+FN}\times 100\%$$

(3) $$F1=\frac{2PR}{P+R}\frac{2TP}{2TP+FP+FN}\times 100\%$$

(4) $$Accuracy=\frac{TP+TN}{TP+TN+FP+FN}\times 100\%$$

where *P*, *R* and F1 represent the precision, recall and F1-score performance metrics for each computational method. The accuracy metric considers all the positive recognition outputs achieved by the ML methods from all the samples used for the testing process for each ML method.

#### 3.5.4. Optimisation Process

The grid search approach is used in this work to optimise the parameters of the computational methods [48,49]. The optimisation process uses data samples from the test dataset. This optimisation process involves the following steps. First, cross-validation, dimensionality reduction and the machine learning method are integrated into a pipeline. Second, a coarse grid search covering the whole search space to locate the region that provides a high accuracy is implemented. Third, a more detailed grid search targeting the region found in the previous step is applied. The coarse and detailed grid search steps are repeated until the accuracy value achieved does not change or remains stable. Fourth, the final parameters obtained from the iterative optimisation process are used in the ML pipeline for the recognition process in testing mode but the optimisation process is disabled. The optimised parameters change for each ML method. For example, in Decision Trees, optimisation is applied to the number of branches in the tree, the number of sample data per branch, and the number of features per branch. In Random Forest, optimisation is applied to the number of individual Decision Trees in the forest. In KNN, optimisation is applied to the number of neighbours and the weights. In MLP, optimisation is applied to the number of hidden layers and neurons, the activation function, the learning rate and the epochs. In CNNs, optimisation is applied to the kernel size, filters, activation function, and epochs. In SVM, optimisation is applied to the kernel and regularisation parameter. Finally, the recognition outputs from this last process are used to compute the performance metrics for evaluation of the ML methods. This optimisation process is applied to each of the ML approaches implemented in this work using raw data and using PCA and LDA dimensionality reduction [44].

## 4. Experiments and Results

**Figure 5:**
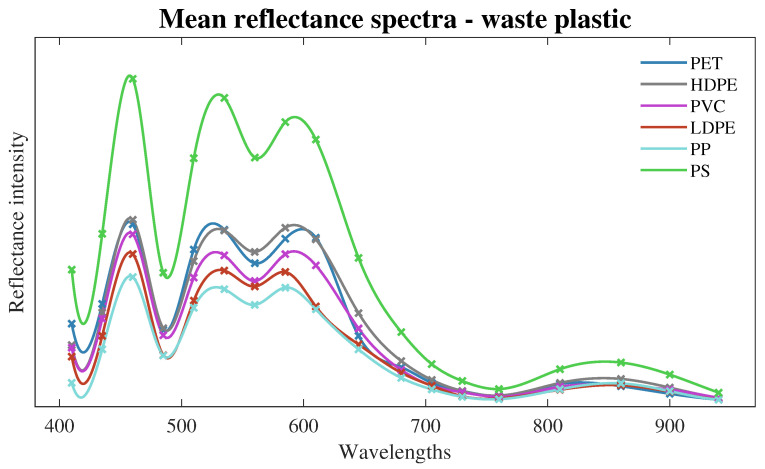
Meandata from plastic waste samples collected in Section 3.4.

In this section, experiments are presented to evaluate the capability of the low-cost spectroscopy sensor for recognition of plastic types using plastic samples from household waste. The experiments for the recognition of plastic types use the dataset and machine learning methods are presented in Section 3.4 and Section 3.5, respectively. The mean or averaged values of the raw data samples collected from the six plastic types are shown in Figure 5. This plot is only for visual inspection purposes, but for the training and testing processes, raw data from the spectroscopy sensor are employed. From visual inspection of this plot, the PS plastic type seems to be more accurately identified over the other plastics. However, the use of machine learning can help to identify the six types of plastic being measured by the spectroscopy sensor, as shown in the next paragraphs.

The results for the recognition of plastic waste are presented in terms of the accuracy, precision, recall and *F*1-score. All computational methods implemented in this work (MLP, CNN, SVM, Decision Trees, Random Forest, AdaBoost, kNN, Logistic Regresion, LDA and Naive Bayes) were trained, optimised and tested using the sensor data collected from household plastic waste shown in Figure 1b and Figure 3a–e. The experiments for the recognition of plastics with each computational method were implemented using input data in three different configurations: (1) raw sensor data as input to the ML method, (2) data preprocessed using PCA followed by the ML method and (3) data preprocessed using LDA followed by the ML method. This approach for the input to the ML methods is used to evaluate and compare the impact of dimensionality reduction in the plastic recognition process.

**Figure 6:**
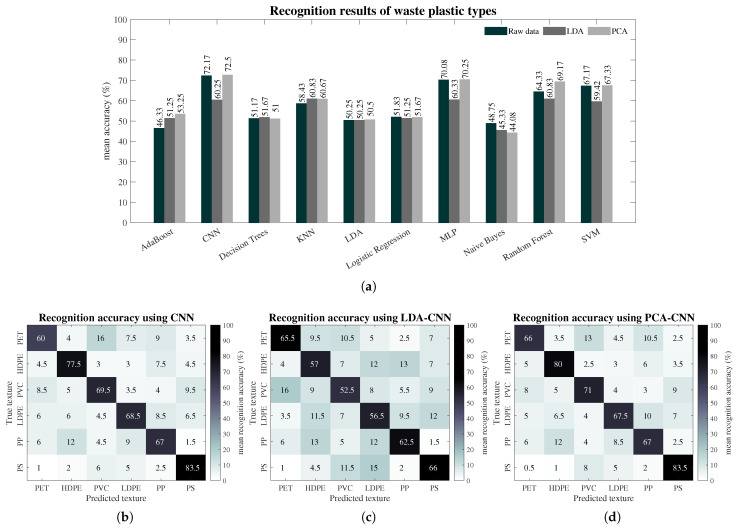
Results from plastic waste and each computational method. (**a**) Mean recognition accuracy from each machine learning method using (i) raw data only, (ii) PCA preprocessing and (iii) LDA preprocessing. (**b**–**d**) Confusion matrices with the highest recognition accuracy for each plastic type using machine learning and raw data, LDA and PCA.

The mean recognition accuracy results over all plastic types and for each computational method are shown by the bar plot in Figure 6a. These results are obtained by computing the mean from the 10 folds used in the cross-validation approach. The four best recognition performances were achieved by the use of (1) PCA for dimensionality reduction followed by a Convolutional Neural Network (PCA–CNN), (2) PCA followed by Multi-Layer Perceptron (PCA–MLP), (3) PCA followed by Random Forest (PCA–Random Forest) and (4) PCA followed by Support Vector Machine (PCA–SVM), with mean accuracies over all plastic types of 72.5%, 70.25%, 69.17% and 67.20%, respectively. The bar plot shows that the majority of computational methods are able to achieve accuracies of over 50% using raw data only, but the results are still improved by preprocessing the data with PCA. LDA for data preprocessing also shows slight improvements over raw data and PCA when it is used together with the Decision Tree method (LDA–Decision Trees) and the k-Nearest Neighbour method (LDA–kNN), achieving accuracies of 51.67% and 60.83%, respectively. The only cases where raw data alone were able to achieve better accuracies (not preprocessed by PCA and LDA) are with Logistic Regression (51.83% accuracy) and Naive Bayes (48.75% accuracy) computational methods.

The confusion matrices in Figure 6 show the recognition accuracy of each individual plastic type achieved by the CNN with preprocessing stages, which presented the best performance. The accuracy values in these confusion matrices were obtained using Equation (4) with information from *TP*, *FP*, *TN* and *FN* values. The results from PCA–CNN in the confusion matrix of Figure 6d show that all plastic types are recognised, with accuracies ranging from a minimum of 66% for PET to the highest accuracy of 83.5% for PS. The CNN with raw data alone, shown in Figure 6b, achieved minimum and maximum accuracies of 60% and 83.5% for PET and PS, respectively. The performance of the LDA–CNN configuration drops to a minimum accuracy of 52.5% for PVC and a maximum accuracy of 66% for PS plastic, as shown in Figure 6c. These confusion matrices also indicate the level of confusion between all plastics for the CNN method. In the matrix shown in Figure 6b, using raw data alone with CNN method, the largest errors are presented for PET being confused with PVC, HDPE with PP, PVC with PET, LDPE with PP, PP with PET and PS with PVC. For the case of LDA and the CNN in Figure 6c, the largest confusion between plastics is PET being confused with PVC, HDPE with PP, PVC with PS, LDPE with PS, PP with HDPE and PS with LDPE. The largest errors observed for PCA–CNN, which achieved the best recognition performance, are for PET plastic being confused with PVC, HDPE with PP, PVC with PET, LDPE with PP, PP with PET and PS with PVC. These results indicate that for the three configurations (CNN, LDA–CNN, PCA–CNN), PET is largely confused with PVC and HDPE is confused with PP.

Table 1 presents the summary of the recognition performance, including the accuracy, precision, recall and *F*1-score, from the best computational methods that achieved at least a 70% recognition accuracy with data from plastic waste.

## 5. Discussion

This research has presented an analysis and the potential of a low-cost multi-spectra triad spectroscopy sensor module, together with a set of machine learning methods, for the recognition of plastics, including PET (code 1), HDPE (code 2), PVC (code 3), LDPE (code 4), PP (code 5), and PS (code 6).

The experimental setup developed for this study allowed for systematic data collection from household plastic waste. A dataset composed of 1200 samples from plastic waste was collected for subsequent analysis. This dataset is larger than those used for plastic recognition using spectroscopy sensors [11,12,13,36]. Each measurement collected from each plastic sample is composed of 18 wavelengths ranging from 410 nm to 940 nm. Thus, this database offers an alternative source of information for systematic recognition of plastic waste. Moreover, the approach followed in this work, based on the sensor module and experimental setup, allows researchers to replicate the data collection process.

The data from plastic waste were analysed by the set of machine learning methods and the following three approaches: (1) the ML method with raw data only (2) the ML method with data preprocessed using PCA and (3) the ML method with data preprocessed using LDA. PCA and LDA techniques were used for dimensionality reduction and their impacts were observed in the recognition process. The computational methods for the recognition of plastics, implemented in the generalised pipeline shown in Section 3.5.2, were optimised using a grid search approach. The outputs from the identification of plastics showed that the use of PCA for data preprocessing improves the recognition accuracy in most ML methods, particularly for the CNN, MLP, Random Forest, AdaBoost, LDA and SVM, with the highest accuracy of 72.50% achieved by the PCA–CNN configuration. The results also showed that the use of LDA for data preprocessing achieves the highest accuracy of 60.50% when used together with kNN. The only case when using raw data outperformed the use of PCA and LDA was observed with the Logistic Regression approach, with an accuracy of 51.5%. The positive impact when using PCA with ML for plastic recognition aligns with previous works [11]. Even though previous works [13,50] have achieved recognition accuracies ranging from 80.56% to 100%, they employed highly expensive spectroscopy sensors (>GBP 25,000). Additionally, some of these methods focused only on a reduced number of plastic types and excluded PET [11], which tends to create more confusion in the recognition process. In contrast, our proposed system combines the benefits of low-cost multi-spectra NIR sensors (∼GBP 56), dimensionality reduction and ML methods to achieve a reasonable recognition accuracy (72.5%). The results from the recognition of plastic waste with the proposed approach also showed that PET achieved the lowest accuracy of 66% among all plastic types, while the highest accuracy was achieved by PS, with 83.50%. Table 2 shows a comparison, in terms of plastic types, accuracy and cost, of our approach with the highly specialised devices used in the literature for the recognition of plastics. In Table 2, some studies have achieved a high accuracy (>90%), but these works required specialised sensors with costs of >GBP 6k. Some of these sensors are relatively small, but not small enough to embedded them into portable and lightweight tools. In contrast, the triad spectroscopy sensor used in our work is small enough to mount it in lightweight and portable tools and devices such as smart bins, conveyors, wands and others. In general, the higher the recognition accuracy, the better the feasibility for practical applications in real time. However, achieving a 100% accuracy with affordable sensors is a difficult task, but based on the literature, a recognition accuracy above 90% is feasible for practical applications. Therefore, in our work, we researched the potential of the low-cost triad spectroscopy sensor (with a cost of ∼GBP 56) with machine learning for the recognition of plastic types, and even though the highest accuracy achieved was 72.50%, this initial investigation provides reliable and robust results that can be used as a reference for the design and implementation of new/emerging ML methods for the recognition of plastics in offline and real-time modes.

In general, the results show that the proposed approach can identify plastic types using the low-cost triad spectroscopy sensor together with ML methods. Even though the accuracy achieved is not as high as the accuracy reported by expensive spectroscopy sensors, this work offers a reliable, affordable and portable solution that contributes to a sustainable circular economy. Furthermore, this work contributes to addressing the growing plastic waste and pollution emergency found throughout the world.

## 6. Conclusions

This work presented an alternative low-cost approach for the recognition of plastic types (PET, HDPE, PVC, LDPE, PP, PS) commonly found in household waste. This approach exploited the potential of multi-spectral NIR sensors (a triad spectroscopy sensor) together with machine learning methods (MLP, CNNs, Random Forest, Decision Trees, AdaBoost, kNN, LDA, SVM, Logistic Regression, Naive Bayes) within a systematic experimental setup. The data analysis also employed a dimensionality reduction stage with PCA and LDA methods. The results showed that PCA combined with a CNN achieved the best mean accuracy of 72.5% for the recognition of plastics. The results suggest that the proposed approach can identify plastic waste with a reasonable accuracy given the capabilities of the low-cost sensor. Overall, this approach is suitable for the development of material identification systems, contributing to affordable device development for sustainable environments and a circular economy.

## Figures and Tables

**Table 1:** Summary of performance of the best computational methods for recognition of plastic waste.

Best Classifier	Dimensionality Reduction	Accuracy	Precision	Recall	*F*1-Score
CNN	PCA	72.50%	72.43%	72.50%	72.38%
CNN	Raw data	72.17%	72.08%	72.17%	72.03%
MLP	PCA	70.25%	70.24%	70.25%	70.18%
MLP	Raw data	70.08%	70.03%	70.08%	70.00%

**Table 2:** Comparison of accuracy and cost of our approach against highly specialised and costly sensors in related works.

Authors	Sensor	Plastic Types	Number of Samples	Accuracy	Method	Sensor Cost
[11]	Ocean Optics–NIR512	HDPE, LDPE, PC, PS, PET, PVC	184 (Waste)	95.7%	PCA–SVM	>GBP 25,000
[13]	VIAVI—MicroNIR	PE, PP, PVC, PET, PS	250 (Waste)	99%	PLS–DA	>GBP 8500
[12]	Ocean Optics—Flame NIR	PET, HDPE, PVC, LDPE, PP & PS	180 (Waste)	91%	LDA	∼GBP 6000
[36]	ASD Field Spec 4	ABS, PS	26 (Waste)	80.56%	PLS–DA	∼GBP 52,000
This work	Triad Spectroscopy Sensor module	PET, HDPE, PVC, LDPE, PP & PS	423 (Waste)	72.5%	PCA–CNN	∼GBP 56

## Data Availability

Data are contained within the article.
